# *Uncaria tomentosa* Exerts Extensive Anti-Neoplastic Effects against the Walker-256 Tumour by Modulating Oxidative Stress and Not by Alkaloid Activity

**DOI:** 10.1371/journal.pone.0054618

**Published:** 2013-02-07

**Authors:** Arturo Alejandro Dreifuss, Amanda Leite Bastos-Pereira, Isabella Aviles Fabossi, Francislaine Aparecida dos Reis Lívero, Aline Maria Stolf, Carlos Eduardo Alves de Souza, Liana de Oliveira Gomes, Rodrigo Polimeni Constantin, Aline Emmer Ferreira Furman, Regiane Lauriano Batista Strapasson, Simone Teixeira, Aleksander Roberto Zampronio, Marcelo Nicolás Muscará, Maria Elida Alves Stefanello, Alexandra Acco

**Affiliations:** 1 Pharmacology Department, Federal University of Paraná, Brazil; 2 Biochemistry Department, University of Maringá, Brazil; 3 Training Laboratory, Pharmacy Department, Federal University of Paraná, Brazil; 4 Chemistry Department, Federal University of Paraná, Brazil; 5 Pharmacology Department, University of São Paulo, Brazil; University of Kentucky, United States of America

## Abstract

This study aimed to compare the anti-neoplastic effects of an *Uncaria tomentosa* (UT) brute hydroethanolic (BHE) extract with those of two fractions derived from it. These fractions are choroformic (CHCl_3_) and *n*-butanolic (BuOH), rich in pentacyclic oxindole alkaloids (POA) and antioxidant substances, respectively. The cancer model was the subcutaneous inoculation of Walker-256 tumour cells in the pelvic limb of male Wistar rat. Subsequently to the inoculation, gavage with BHE extract (50 mg.kg^−1^) or its fractions (as per the yield of the fractioning process) or vehicle (Control) was performed during 14 days. Baseline values, corresponding to individuals without tumour or treatment with UT, were also included. After treatment, tumour volume and mass, plasma biochemistry, oxidative stress in liver and tumour, TNF-α level in liver and tumour homogenates, and survival rates were analysed. Both the BHE extract and its BuOH fraction successfully reduced tumour weight and volume, and modulated anti-oxidant systems. The hepatic TNF-α level indicated a greater effect from the BHE extract as compared to its BuOH fraction. Importantly, both the BHE extract and its BuOH fraction increased the survival time of the tumour-bearing animals. Inversely, the CHCl_3_ fraction was ineffective. These data represent an in vivo demonstration of the importance of the modulation of oxidative stress as part of the anti-neoplastic activity of UT, as well as constitute evidence of the lack of activity of isolated POAs in the primary tumour of this tumour lineage. These effects are possibly resulting from a synergic combination of substances, most of them with antioxidant properties.

## Introduction

*Uncaria tomentosa* is a vine native to the Peruvian Amazon Basin [1] that is biologically active especially as antiinflammatory, immunonomodulant and anti-oxidant agent. For the last couple of decades, researchers have experimented several types and methods of extraction to observe a wide array of pharmacological properties, including limitation of the epithelial cell death in response to oxidant stress [2], amelioration of the oedema via inhibition of cyclooxygenase-1 and −2 [3], cytoprotection by means of free radical scavenging, reduction of oxidative stress and direct inhibition of the TNF-α production [4]–[6], to name a few.

Most of these researchers have attributed the biological effects of *U. tomentosa* to the pentacyclic oxindole alkaloids present in this plant. This idea is supported by the apparently restricted occurrence of these alkaloids within this genus [7], as well as by studies performed with alkaloids isolated from the plant rather than with brute extracts. Indeed, reference to the use of alkaloids isolated from *U. tomentosa* goes as far back as to 1985, when Wagner and colleagues [8] observed that four out of six oxindole alkaloids present in this plant caused a pronounced enhancement of phagocytosis, both *in vitro* and *in vivo*. More recently, studies with several of these isolated alkaloids yielded reports on their antioxidant, immunomodulant [9] and even anti-neoplastic properties [10], [11]. Other studies, however, have demonstrated that compounds different than the oxindole alkaloids may be at least partially responsible for the observed effects. Among the most cited such substances are triterpenes [12], quinovic acid glycosides [13], and others hitherto not identified [14]. Yet a third hypothesis, albeit older, suggests that the anti-inflammatory properties of UT may be related to a synergic combination of compounds [7], [15].

Currently, growing attention is being paid to the anti-neoplastic potential of *U. tomentosa*. Indeed, various extracts and compounds derived from this plant have been found to alter or downright inhibit the growth and proliferation of several different tumour lineages including human neuroblastoma and glioma [11], HL60 promyelocytic leukaemia [16], [17] and MCF7 breast cancer [18], among others. Also, our team of researchers published one of the first studies of the anti-neoplastic properties of *U. tomentosa* on a solid tumour *in vivo*. In addition to observing an important reduction of the tumour mass and volume as a result of the treatment with a hydroethanolic extract of the plant, we noted a remarkable modulation of the oxidative stress caused by the neoplastic process, both in the liver and in the tumour of the subjects [19]. Our conclusion was that the modulation of the redox processes would probably play a pivotal role in the anti-proliferative effects of the plant, perhaps via alteration of one or more metabolic pathways.

It is only logical to assume that all these anti-neoplastic properties should be due to the pentacyclic oxindole alkaloids, which have been shown to exert the anti-inflammatory properties mentioned above, especially when considering cancer as a chronic inflammatory disease [20], [21]. Thus, the present study aimed to evaluate the anti-neoplastic effects of two different fractions obtained from a hydroethanolic extract of *U. tomentosa*: one composed roughly of pentacyclic oxindole alkaloids and triterpene glycosides (chloroformic fraction, CHCl_3_), and the other composed of most of the substances other than alkaloids present in the original extract, namely, phenolic glycosides and other anti-oxidant substances (*n*-butanolic fraction, BuOH). Additionally, we compared the effects of both fractions to those of the original hydroethanolic extract. The chosen *in vivo* tumour model was the Walker-256 (W256) carcinosarcoma in rats, which was the same model used in our previous experiments.

## Materials and Methods

### Botanic Material, Extraction and Chemical Analyses

All botanic material was kindly provided by the Peruvian Heritage SAC. It consisted on a brute hydroethanolic (BHE) extract of the bark of *U. tomentosa* prepared by decoction using a mixture of ethanol and water in the proportion of 7∶3 for one hour at 20°C, and subsequently dried by atomization until the obtaining of a fine powder. Total alkaloid content was ascertained at 5.03% by means of high-performance liquid chromatography (HPLC) according to methods described elsewhere [22].

To achieve further fractionation, 20 g of this BHE extract were dissolved in ethanol-H_2_O 1∶1 and successively extracted with chloroform and *n*-butanol (3×150 mL for each solvent). After solvent removal under reduced pressure, the fractions in chloroform (labelled CHCl_3_) and butanol (labelled BuOH) were obtained, with a yield of 1.9 and 9.5 g, respectively. These fractions were examined by analytical thin layer chromatography (TLC) using silica gel 60 PF_254_ precoated plates (Whatman) and several solvent systems. The spots were revealed by exposure under UV_254/366_ light, spraying with the Dragendorff alkaloid-marking reagent and with 5% (v/v) H_2_SO_4_ in ethanol (EtOH) solution, followed by heating on a hot plate.

HPLC fingerprints analyses of the CHCl_3_ and BuOH fractions were performed on a Waters HPLC equipped with a 2998 photodiode array detector. For all analyses a Waters X-Terra C18 column (250×4.6 mm, particle size 5 υm) was used. The CHCl_3_ fraction was analysed according the method proposed by Ganzera *et al.* [23]. The eluent for the BuOH fraction consisted of H_2_O with 1% of acetic acid (A) and methanol (B), applied in a linear gradient from 80∶20 (A:B) to 100 (B) over 70 min at a flow rate of 1 mL.min^−1^. The column effluent was monitored at 254 nm.

Nuclear magnetic resonance (NMR) spectra of the CHCl_3_ and BuOH fractions were recorded on a Bruker AC 200 and/or Avance 400 spectrometer, observing ^1^H at 200 or 400 MHz and ^13^C at 50 or 100 MHz. The solvent used was DMSO-D_6_, with tetramethylsilane (TMS) as internal reference.

### *In vitro* Free Radical-scavenging Activity

The reactivity of the *U. tomentosa* BHE extract and both its resulting fractions with the stable free radical 2,2-diphenyl-1-picrylhydrazyl (DPPH) was measured by means of an adaptation of the method of Chen and colleagues [24]. The system consisted of 250 µL of a methanolic solution of DPPH (1 mg in 25 mL), which was then combined with 750 µL of six crescent test solutions of each product. For the BHE extract we tested concentrations within the interval of 1–300 µg.mL^−1^, whereas for its fractions we tested extrapolated concentrations as per the yield of the fractioning process. The decrease in absorbance was measured after 5 minutes. A solution of the reducing agent ascorbic acid (50 µg.mL^−1^) was used as positive control and distilled water was used as negative control. Considering possible colour shifts in the solutions due to naturally occurring pigments in the tested substances, we included measurements of the absorbance of each solution prior to the addition of DPPH, which were subtracted when calculating the final values.

### Tumour Cells, Handling and Inoculation

The Walker-256 tumour was first discovered as a spontaneous carcinosarcoma in the mammary gland of a pregnant rat [25]. The matrixes employed in the present work were kindly donated by Prof. Dr. Luiz Cláudio Fernandes, from the Department of Physiology of the Federal University of Paraná and, originally, by Prof. Dr. Rui Curi, from the Department of Physiology and Biophysics of the University of São Paulo. Extensive studies have already been performed with cells from these sources [26], [27]. Their maintenance was carried out by weekly passages through intraperitoneal (IP) inoculation according to Vicentino and colleagues [28]. After five to seven days, the animal would appear emaciated and with vast ascitic signs. At this point, it was submitted to euthanasia and its ascitic fluid collected and centrifuged for 10 min at 1126 ×*g* at 4°C. The supernatant was discarded, and the precipitate was re-suspended in 1.0 mL of PBS (16.5 mM of phosphate, 137 mM of NaCl and 2.7 mM of KCl). The viability of tumour cells was assessed by the Trypan blue exclusion method in a Neubauer chamber. Finally, around 10^7^ Walker-256 cells were injected subcutaneously in the right pelvic limb of each animal.

### Animal Handling, Experimental Design, Sample Collection and Ethical Issues

Male Wistar rats weighing 180–250 g were obtained from the Central Animal House of the Federal University of Paraná (Curitiba, Brazil). Housing conditions included temperature of 22±1°C, constant 12-hour light-dark cycles and free access to standard laboratory food (Nuvital®) and tap water. No other experiments were conducted in these animals prior to those of the current research.

We performed a pre-clinical trial in this animal model. Treatment began 1 day after the subcutaneous inoculation of 10^7^ tumour cells, and continued for 14 days. All three treatment products were dissolved daily in 6 mL of vehicle (distilled water containing 2% Tween 80). The selected doses, which were administered daily by gavage, were 50 mg.kg^−1^ for the BHE extract (Group BHE) and extrapolated doses for both fractions as per the yield of the fractioning process (Groups BuOH and CHCl_3_). Animals of the control group (Group C) received a similar volume of vehicle. For some parameters, a Baseline group (Group B) was added, which was composed of non-tumour-bearing individuals that received vehicle during 14 days. The number of individuals (*n*) in each of these groups was 4–6.

Following the 14-day treatment, all animals were anesthetized with intraperitoneal injection of ketamine (Quetamina®, Vetnil, Louveira, Brazil), in a dose of 60 mg.kg^−1^, and xylazine (Kensol®, König, Santana de Parnaíba, Brazil), in a dose of 7.5 mg.kg^−1^. Blood samples from the inferior cava vein were obtained for biochemical assays; subsequently, animals underwent euthanasia by diaphragmatic puncture. All primary tumours were removed and had their biophysical parameters measured as described below. Finally, tumour and liver samples were collected and stored at −70°C for further analyses.

All experimental protocols using animals were performed following the recommendations of Brazilian Law 6638 (05/11/1979) for the scientific management of animals and the “Principles of Laboratory Animal Care” (NIH Publication 85 - 23, revised in 1985). The Institutional Animal Ethics Committee of Federal University of Paraná revised and approved all procedures of this study, issuing the certificate number 501.

### Biophysical Parameters Measurement

All animals had their weight measured every other day during the treatment period. After fourteen days, all tumours were removed and weighted in an analytical balance. Tumour volume was calculated by means of the measure of its diameters, according to Mizuno *et al*. [29], using the formula *V (cm3) = 4π/3.a2.(b/2)*, where *a* is the lesser diameter and *b* is the greater diameter, in cm. Furthermore, we assessed the inhibitory effect, when appropriate, using the following formula: *Tumour Suppression (%) = (1 - T/C)*, where *T* is the average of the volumes of the tested group and *C* is the average volume of the control group.

### Plasma Biochemical Assays

Plasma samples were obtained after blood centrifugation at 3000 ×*g* for 10 minutes. These samples were used for determination of plasmatic urea, alanine aminotransferase (ALT) and aspartate aminotransferase (AST) by means of commercial kits (Labtest Diagnostica®, Lagoa Santa, Brazil) using the automating device Cobas Mira, of Roche Diagnostics.

### Oxidative Stress Parameters

- **Determination of the lipid peroxidation rate:** Lipid peroxidation (LPO) rate was measured by the FOX-2 method [30]. This technique determines lipid hydroperoxide synthesis during peroxidation. Tumour and liver samples were homogenized at 25,000 rpm and then dissolved in methanol (1∶2 ratio). Finally, they were centrifuged at 5000 ×*g* for 5 minutes at 4°C. The absorbance of the supernatant was measured at 560 nm in a spectrophotometer model Ultrospec 4300 pro. The results were expressed in nmol.mg.protein^−1^.
- **Determination of the activity of the enzymes catalase, superoxide dismutase and glutathione-S-transferase:** For the biochemical analyses of these enzymes, liver and tumour samples were homogenized in phosphate buffer at pH 6.5. Catalase (Cat) activity was measured according to Aebi [31]. The reaction was examined at 240 nm in a spectrophotometer during 60 seconds for the liver samples and 180 seconds for the tumour samples. This difference in the time of the reaction was due to the much lower Cat levels in tumour tissue when compared to liver tissue.

Superoxide dismutase (SOD) activity was measured in both tissues by the ability of this enzyme to inhibit pyrogallol auto-oxidation, according to the method described by Gao and colleagues [32]. The reaction was performed in a 96-well microplate and examined at 440 nm. The amount of enzyme that inhibited the reaction by 50% (IC_50_) was defined as one unit of SOD, and the enzyme activity was expressed in units of SOD per milligram of total protein (U SOD.mg protein^−1^).

The activity of glutathione-S-transferase (GST) in the liver was measured following the method of Habig and colleagues [33], which is based on the capacity of this enzyme to conjugate the substrate 2,4-dinitrochlorobenzene (DNCB) with glutathione in its reduced form, forming a tioether that can be measured by the increase in absorbance at 340 nm. Enzyme levels were expressed as nmol.minute^−1^.mg of protein^−1^.

- **Determination of the reduced glutathione levels:** Reduced glutathione (GSH) levels were measured by the method described by Sedlak and Lindsay [34]. Tumour and liver tissue were diluted in phosphate buffer 0.1 M (pH 6.5) in the proportion of 1∶10. Subsequently, 250 µL of the homogenate were mixed with trichloroacetic acid (200 µL of 12.5% purity) and kept in ice for 30 min in order to allow protein precipitation. The supernatant was separated by centrifugation at 13,750 ×*g* for 10 min at 4°C. Then, 30 µL of the clear supernatant was mixed with 270 µL of phosphate buffer 0.1 M (pH 8.5) and 5 µL of 5,5′-dithiobis-(2-nitrobenzoic acid) in methanol. The absorbance of the reaction solution was measured at 415 nm in a microplate reader, using reduced glutathione as external standard.
- **Quantification of proteins:** The quantification of proteins in liver and tumour samples was performed according to the method designed by Bradford [35]. The reaction was examined at 595 nm in a microplate reader, using bovine serum albumin (BSA) as protein standard.

### Western Blot Analysis for SOD-1 and Catalase

Liver and tumour samples were homogenized in 50 mM Tris-HCl, pH7.4, with protease inhibitors, on ice. Colorimetric protein assay was performed according Bradford (1977, Bio Rad kit, USA). Sample homogenate proteins (2,5 and 5 µg/lane to SOD-1 and catalase, respectively) were separated by SDS-PAGE electrophoresis (15% polyacrylamide) according to literature-based methods and electrophoretically transferred to a nitrocellulose membrane by electroblotting (150 mA V, 2 h). After the blockade of non-specific binding sites with 0.2% casein, the membranes were incubated with the primary anti-SOD-1 antibody (sheep, policlonal, Calbiochem Lab., Germany, 1∶2000) or anti-catalase antibody (mouse, monoclonal, Sigma Chem. Co., USA, 1∶3000) overnight at 4°C. The membranes were washed with Tris-buffered saline containing 0.2% Tween 20 and incubated with the secondary antibody horseradish peroxidase-conjugated (rabbit anti-sheep, 1∶4000 to SOD-1 and goat anti-mouse, 1∶3000 to catalase). Immunoreactive bands were detected by chemiluminescence (Immun-Star; SuperSignal West Pico, Thermo Scientific, USA) and their intensities were estimated by densitometric analysis (ChemImager 5500 system, Alpha Innotech Corp., USA). Results were normalized by the bands stained with Ponceau.

### Measurement of the Inflammatory Cytokine TNF-α

The measurement of this cytokine was performed in the liver and tumour homogenates by ELISA method, following the manufacturer’s instructions (R&D Systems). A plate of 96 wells was coated with 100 µL of purified anti-mouse TNF-α antibody (1 µg.mL^−1^) and incubated overnight at 4°C. In the following day, recombinant murine TNF-α standard (31.5–2000 pg.mL^−1^) and samples were added into their corresponding wells and the plate was incubated overnight at 4°C. After this period, 100 µL of biotinylated anti-mouse TNF-α was added to each well in an amount of 50 ng.mL^−1^. The plates were then incubated for 2 h at room temperature. Following the removal of the unbound antibody-enzyme reagent, 100 µL of streptoavidin–horseradish peroxidase (HRP) solution was added to the wells, which were then incubated for 20 min. Afterwards, 100 µL of the substrate solution containing hydrogen peroxide and o-phenylenediamine (OPD; Sigma-Aldrich Corporation) was added to the wells for colour development. The enzyme reaction yields a yellow product that turns orange in the presence of 50 µL of the stop solution (H_2_SO_4_ 1 M), which was added to each well after 25 min. The optical density was determined using a microplate reader at 450 nm.

### Survival Analysis

In order to ascertain the possible influence of the treatment with *U. tomentosa* on the overall survival rate of the tumour-bearing animals, we conducted a survival analysis employing different individuals as those used for all other experiments. Subcutaneous inoculation of the Walker-256 cells and the configuration of treatment groups and doses were performed as previously described. Treatment period lasted 30 days instead of 14 days in order to fully observe the survival times for each treatment group, whereupon all surviving animals were subjected to euthanasia by means of anaesthesia with ketamine and xylazine, as described previously, followed by decapitation.

The Kaplan-Meier statistical method was used, and survival curves were compared using the logrank test. This test generates a *p* value that verifies the null hypothesis, which in turn states that all curves are equal.

Additionally, a correlation was constructed between the survival rate (%) after 30 days of treatment and the tumour weight (g) after 14 days of treatment, which was analysed by linear regression.

### Statistical Analysis

Unless otherwise noted, statistical analyses were performed using one-way analysis of variance (ANOVA) with post hoc Neumann–Keuls multiple range testing, in the Graph Pad Prism program version 5.0. Differences were considered significant when *p*≤0.05.

## Results

### Chemical Analyses Showed Different Compositions of Both *U. tomentosa* BHE-Derived Fractions

TLC analyses showed that the CHCl_3_ and BuOH fractions were mixtures. Compounds were revealed with UV_254_ light and H_2_SO_4_ solution in both fractions, but only the CHCl_3_ fraction provided a positive test with the Dragendorff alkaloid-specific reagent, indicating the presence of these compounds in its composition.

**Figure 1:**
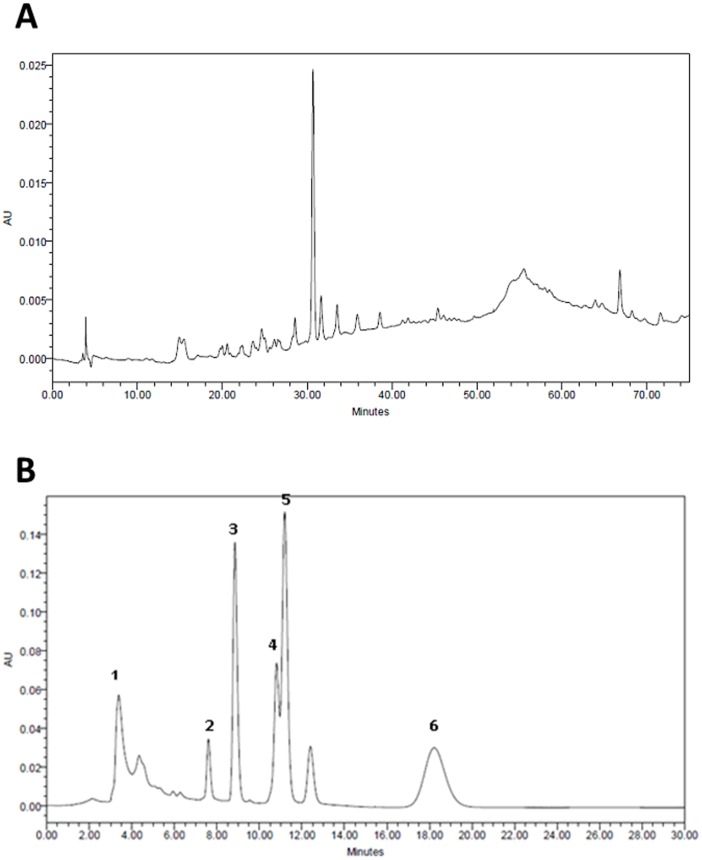
HPLC results. (A) Chromatogram of the BuOH fraction of *U. tomentosa* showing phenolic compounds, including proanthocyanidins in the region between 50 and 60 minutes. (B) Chromatogram of the CHCl_3_ fraction of *U. tomentosa*. The following compounds were tentatively identified as 1- speciophylline; 2- uncarine F; 3- mitraphyline; 4- isomitraphyline; 5- pteropodine and 6- isopteropodine by comparison with data from the literature.

The BuOH fraction was found to be constituted by a complex mixture of components, as shown in its HPLC chromatogram (Figure 1A). Comparison with previously reported HPLC chromatogram of polar extracts of *U. tomentosa* [36] suggests that BuOH fraction contain proanthocyanidins and phenolic compounds, as well as caffeic acid and derivatives.

**Figure 2:**
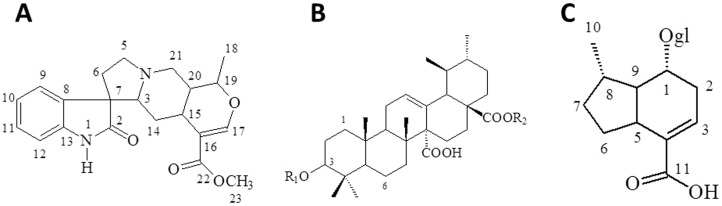
Chemical structures of some of the compounds relevant to this study. (A) Structure of the major alkaloids of *U. tomentosa*. The individual compounds differ in configuration at C-3, C-7, C-15, C-19 and C-20. (B) Structure of quinovic acid (R = H) and its glycosides (R = several sugars), as well as (C) of 7-deoxyloganic acid, all of them isolated from *U. tomentosa*.

The ^1^H NMR spectrum of the BuOH fraction (Figure S1) showed several multiplets at δ 3.0–4.0, along with doublets at δ 4.3–5.5, suggesting the presence of sugars. We also observed signals of aromatic protons (6.5–8.0 ppm) and a broad singlet at δ 8.90, characteristic of a hydroxyl group of phenols or carboxylic acids. Two doublets at δ 7.35 (*J* 1.0 Hz) and δ 5.15 (*J* 5.7 Hz) were associated with 7-deoxyloganic acid [37], which is a rare iridoid illustrated in the figure 2C. In agreement, the ^13^C NMR spectrum of this fraction (Figure S2) showed several peaks assignable to oxygenated carbons (62–82 ppm), anomeric carbons (92–105 ppm), aromatic carbons (115–150 ppm) and carbonyl groups (169 ppm). The presence of 7-deoxyloganic acid was confirmed by correlations observed in the heteronuclear single quantum coherence (HSQC) spectrum (Figure S3) and heteronuclear multiple bond correlation (HMBC) spectrum (Figure S4) experiments. Furthermore, the doublet at δ 5.15 in the ^1^H NMR spectrum showed correlation with a carbon at δ 96.2 (C-1) in the HSQC spectrum and cross-peaks with carbons at δ 33.8 (C-5), 34.8 (C-8), 99.0 (anomeric carbon of glucose) and 150.8 (C-3) in the HMBC spectrum. In addition, the doublet at δ 7.35 showed correlation with a carbon at δ 150.8 (C-3) in the HSQC spectrum and cross-peaks with carbons at d 33.8 (C-5), 96.4 (C-1), 112.1 (C-4) and 169.1 (COOH). In conclusion, the BuOH fraction is constituted of 7-deoxyloganic acid, along with proantocyanidins and phenolic glycosides.

The chromatogram of the CHCl_3_ fraction (Figure 1B) exhibited a profile consistent with the presence of pentacyclic oxindole alkaloids. The compounds were tentatively identified as speciophylline, uncarine F, mitraphyline, isomitraphyline, pteropodine and isopteropodine by comparison with chromatograms previously reported [23] and an analytical report of Peruvian Heritage S.A.C (data not shown). Other unidentified peaks were also observed.

The ^1^H NMR spectrum of the CHCl_3_ fraction (Figure S5) showed signals due the presence of several types of protons, such as aliphatic (0.7–3.0 ppm), oxy-aliphatic (3.0–5.0 ppm), olefinic (5.5 ppm), aromatic (6.8–7.5 ppm) and protons on heteroatoms (10.3–10.5 ppm). Accordingly, the ^13^C NMR spectrum (Figure S6) exhibited more than one hundred peaks, including some signals attributable to carbonyl groups (166–210 ppm). Comparison with spectral data of compounds previously isolated from *U. tomentosa* [13], [38]–[42] led us to conclude that the fraction CHCl_3_ is a complex mixture of oxindole alkaloids and triterpene glycosides. The presence of these classes of compounds was confirmed by detailed analysis of selected regions of the NMR 1D and 2D spectra (Figures S7 and S8), which also revealed the presence of 7-deoxyloganic acid [37]. Thus, the presence of oxindole alkaloids (Figure 2A) was easily confirmed in the ^1^H NMR spectrum by multiplets in δ10.3–10.5, which can be assignable to protons attached to nitrogen. These protons did not show correlations in the HSQC spectrum, but showed cross-peaks with carbons at δ 55.9 and 56.6 (C-7), 133.6 and 133.8 (C-8) and 141.5 and 141.9 (C-13) in the HMBC spectrum. In the ^1^H NMR spectrum we also observed several singlets at δ 0.7–1.0 that are characteristic of triterpenes. In the HSQC spectrum the signals at δ_H_ 0.76, 0.96 and 5.52 showed correlation with signals of carbons at δ_C_ 16.8, 28.1 and 128.5, respectively. The last value suggests a triterpene with an urs-12-ene skeleton, which could correspond to quinovic acid and its derivatives (Figure 2B), which are triterpenes of the oleanane or ursane type that are found free or attached to sugars, and are very common in *U. tomentosa*. HMBC correlations were observed between the proton assignable to H-24 (δ 0.96, s), C-3 (δ 88.4), C-4 (δ 39.1), C-5 (δ 55.6) and C-23 (δ 16.9). The chemical shift of C-3 is compatible with the presence of a sugar attached through an ether linkage in this position. Indeed, a doublet at δ 4.16 (7.2 Hz), which can be assignable to an anomeric proton, showed correlation in the HSQC spectrum with a signal of carbon at δ 105.7 and a cross-peak with C-3 in the HMBC spectrum. Finally, 7-deoxyloganic acid (Figure 2C) was deduced from a doublet at δ 5.15 in the ^1^H NMR spectrum, which showed the same correlations observed in the spectra of the BuOH fraction.

### *U. tomentosa* BHE and its BuOH Fraction Exhibited *in vitro* free Radical-Scavenging Activity

**Figure 3:**
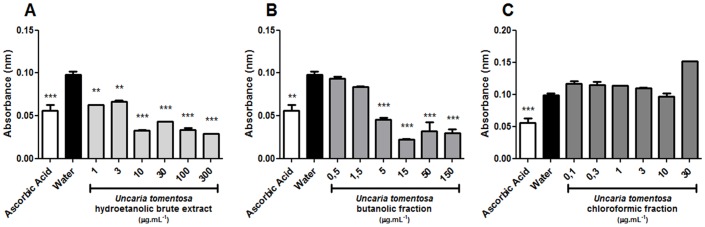
Assessment of the free radical-scavenging activity of the BHE extract of *U. tomentosa* (A), as well as its two resulting fractions: BuOH (B) and CHCl_3_ (C) at various concentrations. Negative control was distilled water and positive control was ascorbic acid (50 µg.mL^−1^). Symbols: **p<0.01 and ***p<0.001 as compared to the negative control.

The antioxidant effect of the BHE extract of *U. tomentosa*, as well as of both its fractions, was assessed *in vitro*. A statistically significant antioxidant activity was observed in all tested concentrations of the BHE extract, this effect being similar to that of the positive control (ascorbic acid). Likewise, the BuOH fraction exhibited satisfactory free radical-scavenging of DPPH starting from the concentration of 5 mg.mL^−1^. However, no tested concentration of the CHCl_3_ fraction was successful in neutralizing DPPH. These results are shown in the Figure 3.

### Both the BHE Extract and its BuOH Fraction Reduced the Tumour Growth

**Figure 4:**
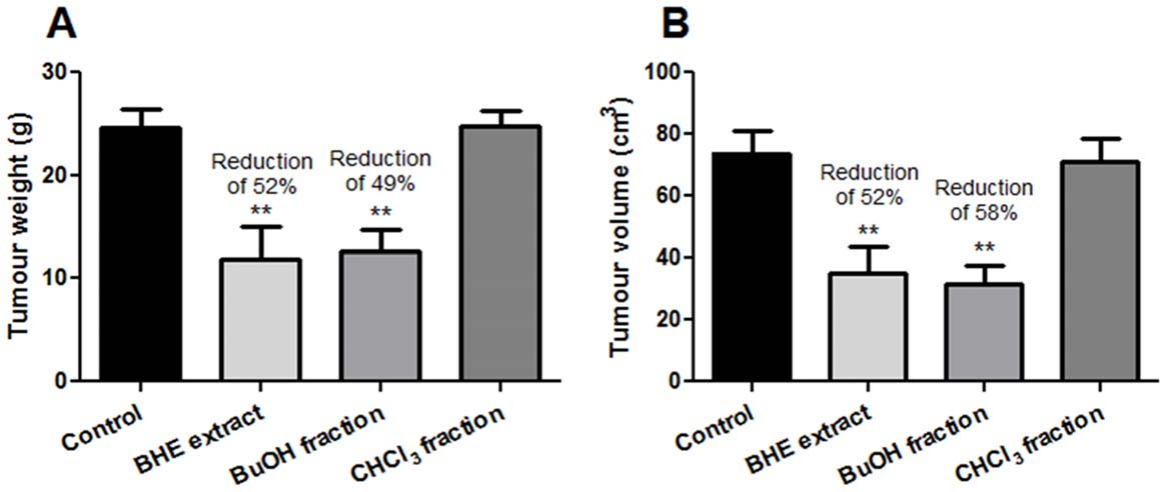
Tumour mass (A) and volume (B) in Walker-256 tumour-bearing rats treated with BHE *Uncaria tomentosa* extract or its two fractions (BuOH and CHCl_3_). Symbols: **p<0.01 as compared to the control group.

To assess the anti-neoplastic effect of *U. tomentosa* we measured some tumour biometric parameters. Tumour mass of the control group at the end of the treatment was 24.67±3.87 g. Treatments with the BHE extract and BuOH fraction had a tumour mass suppression of 52% and 49%, respectively, as compared to control (*p*<0.01). On the other hand, the CHCl_3_ fraction presented a tumour mass of 24.74±3.2 g, which was similar to that of the control group (Figure 4A). Tumour volume results were quite similar to those of tumour mass, exhibiting an evident difference between the control and BHE or BuOH treated groups. The control group showed a tumour volume of 73.8±17.32 cm^3^ at the end of the treatment, while the BHE extract- and BuOH fraction-treated group presented less than 50% of this volume (35.12±16.87 cm^3^ and 31.35±14.93 cm^3^, respectively), both results being statistically significant (*p*<0.01). Still, the group that received the CHCl_3_ fraction presented a tumour volume similar to that of the control group (Figure 4B). The tumour volume suppression rates calculated for the BHE extract and BuOH fraction groups as compared to the control group were 52% and 58% respectively.

### Plasma Levels of ALT and AST were Affected by the Tumour and the Treatments

The plasmatic enzymes ALT and AST were measured as parameters that indicate cellular integrity, especially that of hepatocytes. Statistically significant differences were found for ALT values amongst control and the three treatment groups, however, all the values were found acceptable for rats. Also, treatment with the BHE extract or its BuOH fraction did significantly reduce AST levels (Table 1) in an attempt to reverse the tumour-induced increase of this enzyme. An increase in plasmatic urea was observed in the rats treated with the CHCl_3_ fraction, as compared to baseline, control and BHE extract groups (Table 1).

**Table 1:** Biochemical parameters measured in the plasma of Walker-256 tumour-bearing rats treated during 14 days with BHE *U. tomentosa* extract or its two fractions BuOH and CHCl_3_.

Parameter	Experimental Groups					Units
	Baseline	Control	*Uncaria tomentosa*			
			BHE extract	BuOH fraction	CHCl_3_ fraction	
**ALT**	55.5±5.3	43.4±6.4	44.25±5.1	31.7±10	31.3±5.5	U.L^−1^
**AST**	69.53±4.9 **	324.4±41.2	183.8±60* ##	167.2±69.9* ^###^	288.5±97.9 ^###^	U.L^−1^
**Urea**	40.8±7.5°	34.8±3.4°°	39±3.6°	45.8±4.8	54.5±11.4	mg.dL^−1^

Symbols: *p<0.05 and **p<0.01 as compared to the control group;

## *p*<0.01 and ^###^ *p*<0.001 as compared to the baseline group;

°*p*<0.05 and °°*p*<0.01 as compared to the CHCl_3_ fraction.

### *U. tomentosa* Presented Opposing Activity upon the Oxidative Stress when Comparing Liver and Tumour Results

Having observed a satisfactory antioxidant *in vitro* effect, and considering the different composition of the *U. tomentosa* extract and its fractions, we deemed appropriate to measure oxidative stress parameters *in vivo*, both in hepatic and tumour tissue. Thereby, we assessed the activity of the enzymes superoxide dismutase (SOD), glutathione-S-transferase (GST) and catalase (Cat), along with the levels of reduced glutathione (GSH) and the lipid peroxidation rate (LPO).

**Figure 5:**
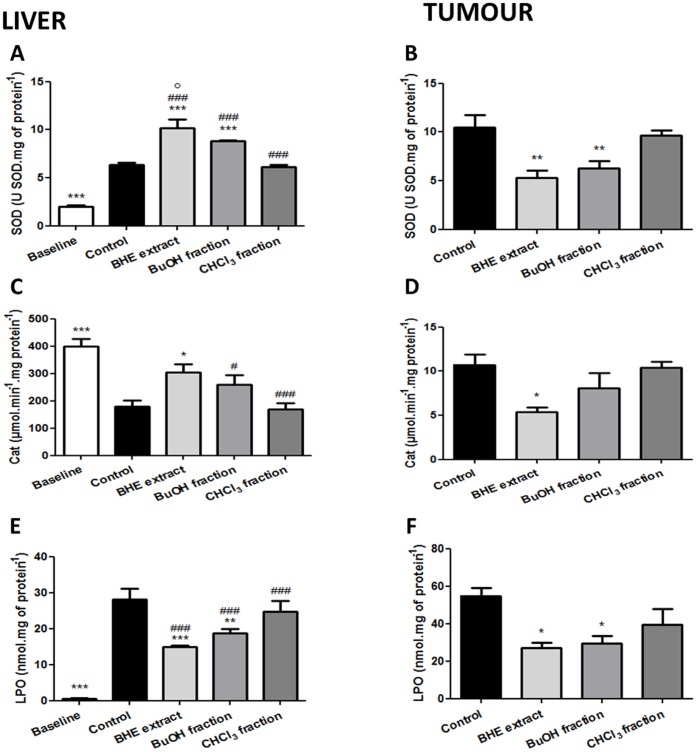
Main oxidative stress parameters measured in W256 tumour-bearing rats treated with *U. tomentosa* BHE extract or its two fractions (BuOH and CHCl_3_). SOD activity in the liver (A) and tumour (B); Cat activity in the liver (C) and tumour (D) and LPO rates in the liver (E) and tumour (F). Symbols: *p<0.05; **p<0.01 and ***p<0.001 as compared to the control group; ^#^ *p*<0.05 and ^###^ *p*<0.001 as compared to the baseline group; °*p*<0.05 as compared to the BuOH fraction.

The experiments regarding SOD activity produced interesting results. In the hepatic tissue of the animals of the control group we found SOD levels of 6.36±0.44 U SOD.mg of protein^−1^, which were quite increased when compared to those of the baseline group (1.99±0.36 U SOD.mg of protein^−1^). Treatment with the BHE extract further increased them to 10.16±1.81 U SOD.mg of protein^−1^ (*p*<0.001) and the BuOH fraction exhibited a significant increase as well, to 8.8±0.28 U SOD.mg of protein^−1^ (*p*<0.001). However, a statistically significant difference was found among these two treatment groups. In opposition, treatment with the CHCl_3_ fraction generated similar SOD values as those of the control group. Remarkably, we obtained opposite results regarding the activity of this enzyme in the tumour tissue. SOD activity in the tumours belonging to the rats of the control group was 10.5±2.89 U SOD.mg of protein^−1^, and treatment with the *U. tomentosa* BHE extract and its BuOH fraction significantly reduced these levels. However, the CHCl_3_ fraction exhibited SOD levels similar to those of the control group. These results are presented in the figures 5A and 5B.

Similar behaviour was observed upon measuring Cat activity. In the liver samples of the control group, this enzyme was found in amount of 179.5±48.92 µmol.min^−1^.mg of protein^−1^, which represents an important decrease when compared to baseline values (399.8±47.65 µmol.min^−1^.mg of protein^−1^). Treatment with the *U. tomentosa* BHE extract increased Cat activity to 303.8±62.37 µmol.min^−1^.mg of protein^−1^ (p<0.05), constituting a full reestablishment of baseline levels as there is no difference between these groups. Nevertheless, both BuOH and CHCl_3_ fractions failed to show a statistically significant increase in Cat activity. Most interestingly, results in the tumours are in opposition of those found in the liver, but with much lower values of Cat activity in this tissue. These results are outlined in the figures 5C and 5D.

Regarding LPO measurements, in the liver samples of the control group we found levels of 28.22±6.7 nmol.mg of protein^−1^. In the same way as what we observed with SOD, treatments with the *U. tomentosa* BHE extract and its BuOH fraction reduced significantly these levels (Figure 5E) in an attempt to return this parameter to baseline levels. As expected, however, the CHCl_3_ fraction showed similar LPO levels as those of the control group (54.8±9.22 nmol.mg of protein^−1^). In the tumour we observed a similar pattern. While treatment with the BHE extract and its BuOH fraction reduced LPO levels in a statistically significant manner, the CHCl_3_ fraction did not alter this parameter (Figure 5F).

No statistically significant differences were found between the experimental groups regarding GST activity and GSH levels in the liver. Yet, treatment with the BHE extract did reduce GSH levels in tumour tissue when compared to the control group (*p*<0.05). Numeric values of these results are presented in the Table 2.

**Table 2:** GSH levels in liver and tumour tissue, as well as GST activity in the liver of W256 tumour-bearing rats treated with *U. tomentosa* BHE extract or its two fractions (BuOH and CHCl_3_).

Parameter	Experimental Groups					Units
	Baseline	Control	*Uncaria tomentosa*			
			BHE extract	BuOH fraction	CHCl_3_ fraction	
**Liver GSH**	589.3±170.2 ^***^	103.0±13.8	109.8±7.82 ###	96.48±6.21 ###	96.57±6.63 ###	nmol.mg ptn^−1^
**Tumour GSH**	(does not apply)	22.44±2.39	13±1.47 ^*^	18.78±6.86	24.8±4.56	nmol.mg ptn^−1^
**Liver GST**	0.6±0,07 ^***^	1.35±0.15	1.24±0.38 ###	1.21±0.24 ###	1.17±0.31 ###	nmol.min^−1^.mg ptn^−1^

Symbols: *p<0.05 and ***p<0.001 as compared to the control group, respectively;

### *p*<0.001 as compared to the baseline group.

### No Differences were Found in Protein Expression of Catalase and SOD-1

**Figure 6:**
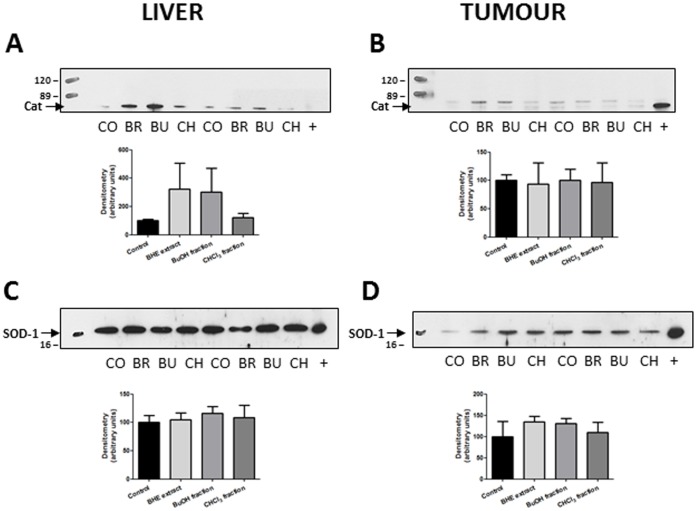
Catalase expression in liver (A) and tumour (B), and SOD-1 expression in liver (C) and tumour (D), as measured by Western blot. The densitometry graphs are displayed beside each picture. Group abbreviations: CO: Control, BR: BHE extract, BU: BuOH fraction, CH: CHCl_3_ fraction.

In view of the interesting divergences in the liver and tumour activity of the *U. tomentosa* BHE extract and its BuOH fraction, we measured the protein expression of catalase and SOD-1 in these tissues by means of Western blot. Despite interesting tendencies regarding hepatic catalase, no statistically significant differences were found among the groups (Figure 6).

### All *U. tomentosa* Treatment Groups Reduced the TNF-α Level in the Liver, however Only the BHE Extract Successfully Reversed it to Baseline Values

**Figure 7:**
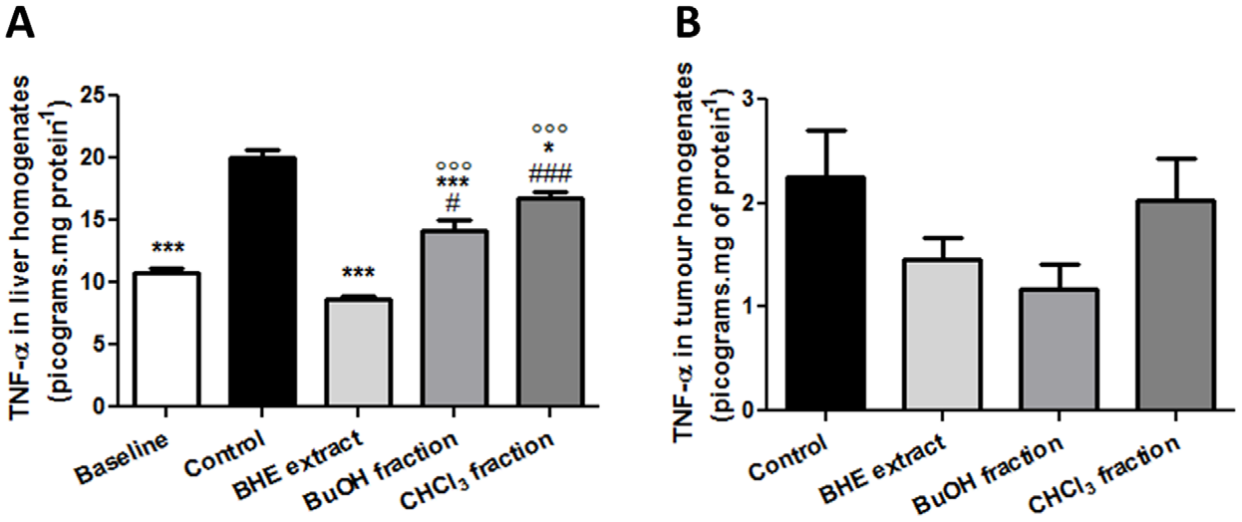
Levels of the inflammatory cytokine TNF-α in the hepatic (A) and tumour (B) homogenates of Walker-256 tumour-bearing rats treated with *U. tomentosa* BHE extract or its two fractions (BuOH and CHCl_3_). Symbols: ***p<0.001 as compared to the control group; ^##^ *p*<0.01 and ^###^ *p*<0.001 as compared to the BHE extract-treated group; °°°p<0.001 as compared to the baseline group.

Since tumour development is characterized by an intense inflammatory reaction, TNF-α levels in the liver and tumour homogenates were measured as an inflammation marker. As the Figure 7 illustrates, the group treated with the BHE extract showed a TNF level of 8.68±0.33 pg.mg of protein^−1^, which is less than half of the value corresponding to the control group: 19.98±1.61 pg.mg of protein^−1^. Furthermore, these levels are similar to those of the Baseline group (10.74±0.91 pg.mg of protein^−1^), constituting an effective reversal to baseline status. Treatment with the BuOH fraction also achieved a statistically significant decrease in TNF-α, yet this decrease was not as pronounced as that of the BHE extract, and there exists statistical difference both between these two groups and between BuOH and Baseline groups. Finally, treatment with the CHCl_3_ fraction achieved a modest TNF-α reduction as compared to Control group: 16.50±2.46 pg.mg of protein^−1^. Despite evident tendencies, no statistically significant differences were found among the experimental groups regarding the TNF-α level on the tumour homogenates.

### Survival Index was Improved by Treatment with *U. tomentosa* BHE Extract and its BuOH Fraction

**Figure 8:**
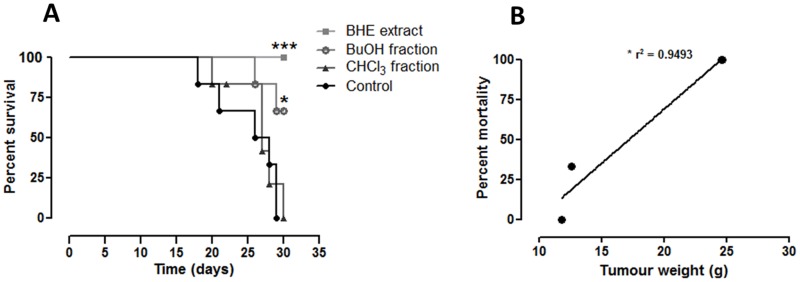
Survival analysis of W256 tumour-bearing rats treated with *U. tomentosa* BHE extract or its two fractions (BuOH and CHCl_3_) during 30 days (A). Data is expressed as percentage of survival as per the logrank (Mantel-Cox) test. (B) Correlation between tumour weight (g) and survival rate (%) analysed by linear regression. Symbols: *p<0.05 and ***p<0.001 as compared to the control group.

Our survival analysis revealed statistically significant differences between the studied groups. While all the tumour-bearing rats treated with the BHE extract survived the entire observation period (30 days), no individual belonging to the control and CHCl_3_ groups remained alive at the end of the trial. Regarding the BuOH fraction, we obtained a survival rate of 66.67%, seeing that two out of six individuals died within the treatment period. These results are illustrated in the figure 8A. A positive correlation was observed between the survival rate and the tumour weight of each tumour-bearing rat after treatment during 14 days (Figure 8B).

## Discussion

*Uncaria tomentosa* has been widely studied and acclaimed as a powerful antioxidant and immunomodulant agent. Currently, considerable attention is being granted to its anti-neoplastic potential as well. Various preparation methods and administration regimes have been tested against tumour lineages *in vitro* [10], [16], [18], [43] and *in vivo* [19], with promising results. Our current efforts follow the wake of some investigators, that experimented with fractions and isolated compounds of *U. tomentosa* extracts with the aim to attribute the observed pharmacological activities to a specific substance or group of substances [10], [11], [17], [44].

Nowadays, it is known that *Uncaria tomentosa* accumulates alkaloids, triterpenes and other classes of compounds, including phenolic glycosides, flavonoids and proanthocyanidins [8], [13], [36], [37], [39]–[41], [45]. Among the most abundant alkaloids found in this plant, there is a mixture of pentacyclic oxindole stereoisomers that differ in configuration at chiral centres in the positions 3, 7, 15, 19 and 20 (Figure 2A). In attempting to explain the anti-neoplastic effects of *U. tomentosa*, researchers have demonstrated interest in this group of substances, based on previous observations that gave them credit for its anti-inflammatory activity [8], [9]. However, it is important not to overlook the fact that cancer is a vast group of diseases that may vary substantially among them. Hence, the number of possible clinical scenarios is equally diverse, with different outcomes among tumour lineages, individuals affected, and even throughout the various locales affected by the neoplastic process in each individual [46].

Pilarski and colleagues [47] demonstrated awareness of this wide range of possible deviations by cross-testing extracts of *U. tomentosa* of different alkaloid content against several tumour lineages, both *in vivo* and *in vitro*. Interestingly, their results suggest that preparations with greater alkaloid concentrations do not necessarily achieve the best anti-neoplastic effects. As possible explanations for their results, the authors suggest an apparent selectivity of the fractions with higher alkaloid levels for some tumour lineages, low water solubility of isolated alkaloids and issues regarding the IC_50_ measures. Taking into account the previous observations of our research team, including the importance of oxidative stress in the W256 *in vivo* tumour model [48]–[50], and the relevant synergism between antioxidant and cytotoxic components in the anti-neoplastic effects of *U. tomentosa* [19], we propose yet another hypothesis: *U. tomentosa* fractions that are alkaloid-rich, but otherwise devoid of most other substances present in the original extract may perhaps leave out and disregard the antioxidant effects of such other substances, along with the beneficial synergic effects that they could be providing. In the present study we sought to put this last hypothesis to the test by evaluating the anti-neoplastic effects of a *U. tomentosa* fraction composed roughly of alkaloids (CHCl_3_) and a fraction composed of most other antioxidant substances (BuOH), finally comparing these fractions to the original BHE extract containing all groups of compounds.

According to our chemical analyses, the CHCl_3_ fraction contains alkaloids, triterpenes and 7-deoxyloganic acid, the latter also found in the BuOH fraction, along with antioxidant substances such as phenolic glycosides and anthocyanidins. These observations are further supported by the DPPH results, which point out the BHE extract as the most effective free radical scavenger. Significant antioxidant activity in the BuOH fraction was detected, while the CHCl_3_ fraction had no antioxidant activity whatsoever. The results of this assay are in accordance to the aforementioned composition of these three substances and are adequate for the testing of our hypothesis.

Expressive reduction in tumour weight and volume was observed in W256 tumour-bearing rats treated with both BHE extract and BuOH fraction. This restriction of tumour development was directly related with increase in the survival rate, as shown in the figure 8B. All (100%) and two thirds (66%) of the rats treated with the BHE extract and BuOH fraction, respectively, survived for the tested period (30 days), while none of the animals treated with the CHCl_3_ fraction nor those who received vehicle (control group) survived. These results clearly show that by fractioning *U. tomentosa* extracts, active compounds that present anti-neoplastic effects are also separated, as well as constitute evidence of the importance of the oxidative stress modulation as part of the action mechanisms of this plant.

The results obtained with the *in vivo* oxidative stress parameters also confirmed these findings. SOD and LPO measures in the liver and tumour tissue demonstrated a major beneficial effect of the *U. tomentosa* BHE extract and BuOH fraction, while the CHCl_3_ fraction consistently produced negligible results. It is noteworthy that the BHE extract was significantly more successful than its BuOH fraction in heightening hepatic SOD levels, which were already increased in the control group as compared to the baseline group as a natural reaction to the considerable oxidative stress caused by the neoplastic process. Regarding Cat measures in both tissues, only the BHE extract achieved a statistically significant effect as compared to control, effectively increasing hepatic Cat levels to similar values as those of the baseline group. Despite the evident lower levels of GSH in tumour tissue found in the BHE extract and BuOH fraction-treated groups as compared to control, only those of the BHE extract were statistically significant. This is a favourable result, taking into account the important role that GSH plays in such cellular processes as the transport of amino acids, the synthesis of proteins and DNA, and even cellular detoxification [51]. It seems appropriate to establish that the biological activities of *U. tomentosa* are indeed enhanced by the synergic action of its various components.

It is remarkable to acknowledge that *U. tomentosa* seems to exert a degree of selectivity over its site of action, an observation which supports our previous findings [19]. Indeed, in various parameters we observed that the BHE extract (and sometimes even its BuOH fraction) had different, if not opposite effects in the liver and tumour tissue. Even more important is the fact that this selectivity resulted in an apparent protection of the hepatocytes, which came along with a simultaneous attack to the neoplastic cells. This most intriguing and favourable behaviour was observed for the SOD enzymatic system, which is responsible for the conversion of the superoxide anion to oxygen and hydrogen peroxide. It was suggested that the Walker-256 tumour-bearing rats present an increased amount of superoxide anion through the respiratory chain [49], promoting an oxidative cascade that could lead to necrosis [52], [53]. Thus, the increase in SOD observed in the hepatic tissue is beneficial, because it suggests an adequate response to this superoxide excess. Conversely, treatment with *U. tomentosa* resulted in a reduction of the SOD levels in the tumour tissue, which in turn constitutes an advantageous result as well, as it indicates that the tumour cells are being left more susceptible to the effects of the superoxide anion. This apparent selectivity was also observed for Cat levels. The importance of Cat in the neoplastic scenarios has long been appreciated, as various studies have demonstrated that increased activity of this enzyme in tumour cells constitute a protection mechanism against cell death induced by reactive oxygen species (ROS), and that by inhibiting this enzyme, the neoplastic cells become once more vulnerable to this outcome [54], [55]. Our findings indicate that Cat levels were reduced in the tumour tissue and increased in the liver, both these results occurring simultaneously and due to the treatment with the *U. tomentosa* BHE extract. Indeed, we may be witnessing a protective effect on the liver associated with a further exposure of the tumour cells to ROS-induced necrosis/apoptosis.

Meaning to further characterize this observation, the protein expressions of both catalase and SOD-1 were assessed by Western blot. As no statistically significant differences were found among the experimental groups, it seems appropriate to assume that the treatments could be increasing the individual output of these enzymes, resulting in a greater overall activity despite equivalent expression rates. In addition to this hypothesis, the lack of differences in the expression of SOD-1 does not exclude an eventual effect upon the expression of the other two isoforms of this enzyme, namely SOD2 (mitochondrial) and SOD3 (extracellular).

According to Valko *et al*. [56], the lower Cat activity induced by various tumours has been attributed to an increase in TNF-α level, which reduces hepatic Cat activity [57], [58]. The BHE extract and its BuOH fraction expressively reduced the TNF-α level on the liver homogenates of tumour-bearing rats when compared to control, perhaps contributing to a recovery of the hepatic Cat activity in these animals. The mensuration of this cytokine in liver homogenates also indicates an anti-inflammatory activity of both the BHE extract and its BuOH fraction. Interestingly, however, we observed an effective reversal of TNF-α to baseline values as a result of the former treatment, leading us to conclude that, regarding this parameter, the BHE extract is indeed more effective than its BuOH fraction. The CHCl_3_ fraction exhibited a mild reduction of this cytokine, which seems natural considering the recognized anti-inflammatory properties of isolated pentacyclic oxindole alkaloids. On the whole, reduction of the cytokine TNF-α is bound to ameliorate some hallmarks of this tumour, such as cachexia [59]. The tumour levels of TNF-α were quite lower when compared to those of the liver. This is probably caused by the histological differences between these tissues; with the latter containing TNF-α-secreting Kupffer cells and the former encompassing mainly fibroblasts that do not secrete TNF-α. Regarding the tumour production of this cytokine, while the same tendencies as those of the liver were observed, no statistical significances were found.

Treatment with the *U. tomentosa* BHE extract and its BuOH fraction successfully reduced the lipid peroxidation (LPO) rates in the tumour as well as in the liver. As we did not observe the aforementioned locale selectivity for this parameter, we can only assume that the substances responsible for that particular behaviour work upon somewhat restricted pathways. Tumour results notwithstanding, the reduction of LPO in the liver may indicate greater protection of the cell membrane integrity, as it is highly susceptible to oxidative stress-induced lipid peroxidation. This particular effect in the liver is further supported by the reduction in plasmatic AST in the individuals treated with both the BHE extract and its BuOH fraction, as compared to control, bringing this parameter closer to baseline values. As this enzyme belongs in the intracellular medium of hepatocytes, high plasmatic levels commonly imply death and lysis of these cells or increase in its membrane permeability at the very least [60]. The unaltered ALT values suggest that the effects of *U. tomentosa* are not restricted to the liver, given that one major difference between both transaminases is that while ALT is restricted to the cytosol of hepatocytes, AST may be found in various other tissues, such as myocardium, skeletal muscle, kidneys, brain, pancreas, lungs and blood cells [61]. The measurement of plasmatic urea did not yield any statistical differences among groups.

On the whole, our results support our initial hypothesis. Numerous substances seem to be acting synergically along with the oxindole alkaloids or even independently of them, even appearing to exert some degree of selectivity upon its locale of action. More studies are required in order to evaluate the degree of participation of these and other substances in the mechanisms by which *U. tomentosa* exerts its anti-neoplastic effects.

## Conclusions

This paper confirms the expressive anti-neoplastic and anti-oxidant activity of *Uncaria tomentosa* preparations. Brought together, our results constitute evidence that the oxindole alkaloids present in this plant are not the sole substances responsible for its biological effects, at least when tested against the primary tumour of the Walker-256 lineage *in vivo*. Modulation of oxidative stress appears to be of utmost importance in thwarting the neoplastic process triggered by this tumour, which is probably achieved by means of a combined and synergic activity from different classes of chemical compounds existing in the brute hydroethanolic extract of this plant. The anti-neoplastic effects produced in this manner seem even more appealing when considering its anti-oxidant and metabolic effects in the liver.

Henceforth, we deem appropriate to perform new experiments, testing the BHE extract and its fractions against different neoplastic scenarios, both *in vitro* and *in vivo*. It would also be interesting to evaluate oxidative stress parameters in other tissues than the liver, in an attempt to further explore the apparent selectivity that *U. tomentosa* seems to exert over its locale of action, and thus achieve a better understanding of the overall therapeutic potential of this plant.

## Supporting Information

Figure S1 **^1^H NMR spectra of the BuOH fraction of** ***U. tomentosa*** **(200 MHz, DMSO-D_6_).**
(TIF)

Figure S2 **^13^C NMR spectrum of the BuOH fraction of** ***U. tomentosa*** **(200 MHz, DMSO-D_6_).**
(TIF)

Figure S3 **HSQC spectra of the BuOH fraction of** ***U. tomentosa*** **(400 MHz, DMSO-D_6_).**
(TIF)

Figure S4 **HMBC spectra of the BuOH fraction of** ***U. tomentosa*** **(400 MHz, DMSO-D_6_).**
(TIF)

Figure S5 **^1^H NMR spectrum of the CHCl_3_ fraction of** ***U. tomentosa*** **(400 MHz, DMSO-D_6_).**
(TIF)

Figure S6 **^13^C NMR spectrum of the CHCl_3_ fraction of** ***U. tomentosa*** **(200 MHz, DMSO-D_6_).**
(TIF)

Figure S7 **HSQC spectra of the CHCl_3_ fraction of** ***U. tomentosa*** **(400 MHz, DMSO-D_6_).**
(TIF)

Figure S8 **HMBC spectra of the CHCl_3_ fraction of** ***U. tomentosa*** **(400 MHz, DMSO-D_6_).**
(TIF)
