# Supplementary figures and images for: Uncaria tomentosa Exerts Extensive Anti-Neoplastic Effects against the Walker-256 Tumour by Modulating Oxidative Stress and Not by Alkaloid Activity
Source: PLoS One. 2013 Feb 7;8(2):e54618. doi: 10.1371/journal.pone.0054618 (PMC3567083; doi:10.1371/journal.pone.0054618)

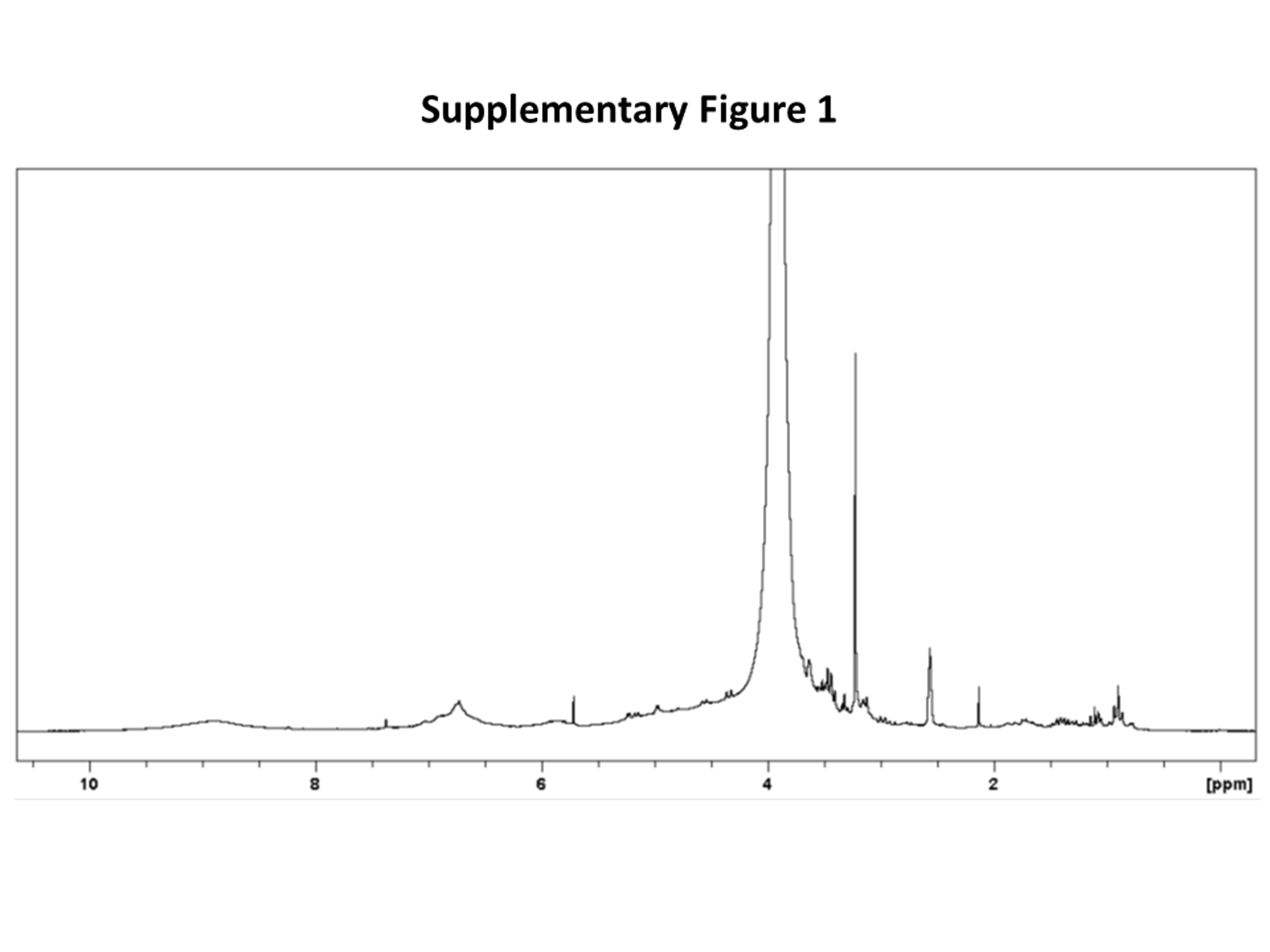

Supplement: Figure S1 — 1H NMR spectra of the BuOH fraction of U. tomentosa (200 MHz, DMSO-D6). (TIF) [file pone.0054618.s001.tif]

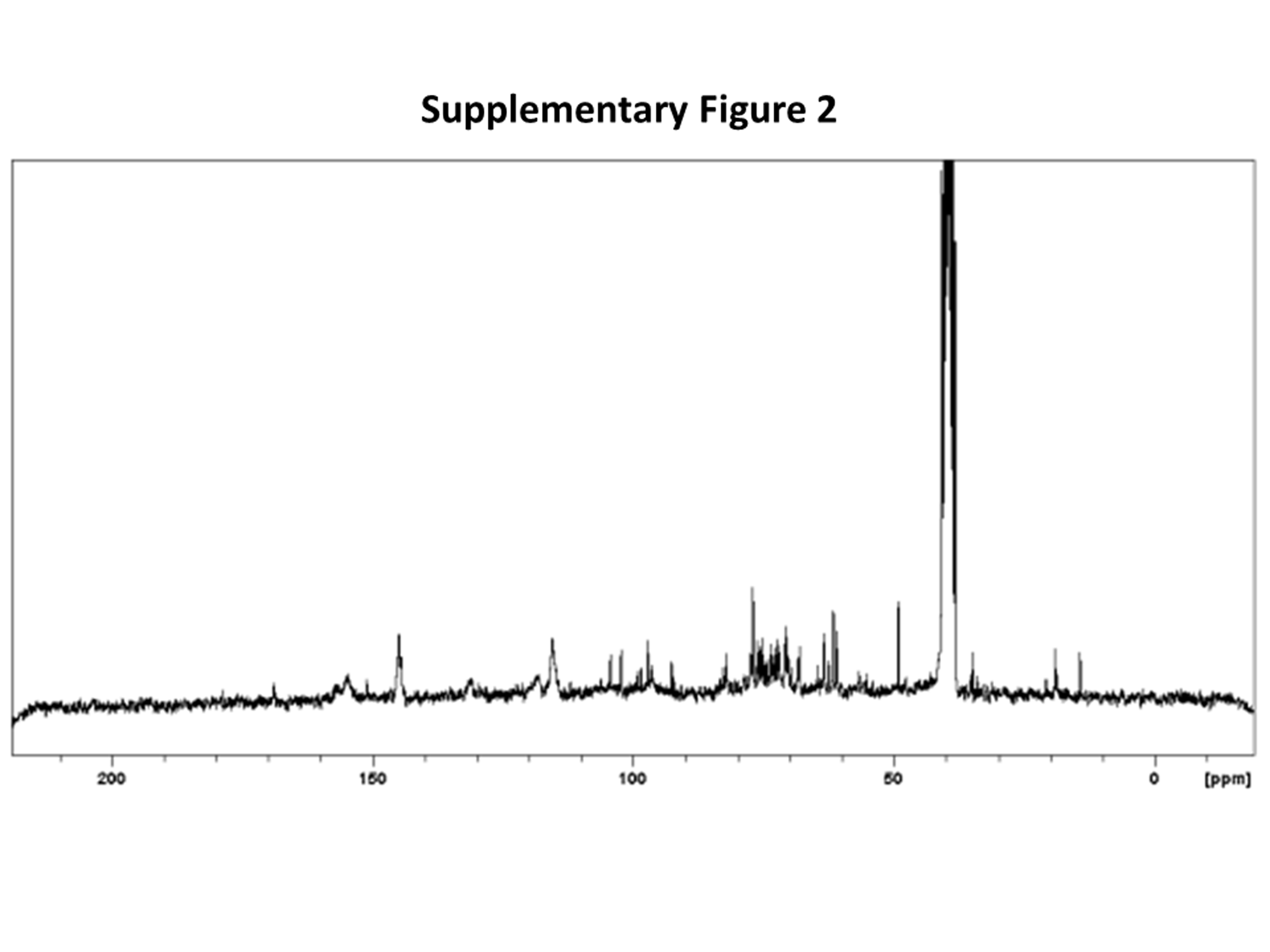

Supplement: Figure S2 — 13C NMR spectrum of the BuOH fraction of U. tomentosa (200 MHz, DMSO-D6). (TIF) [file pone.0054618.s002.tif]

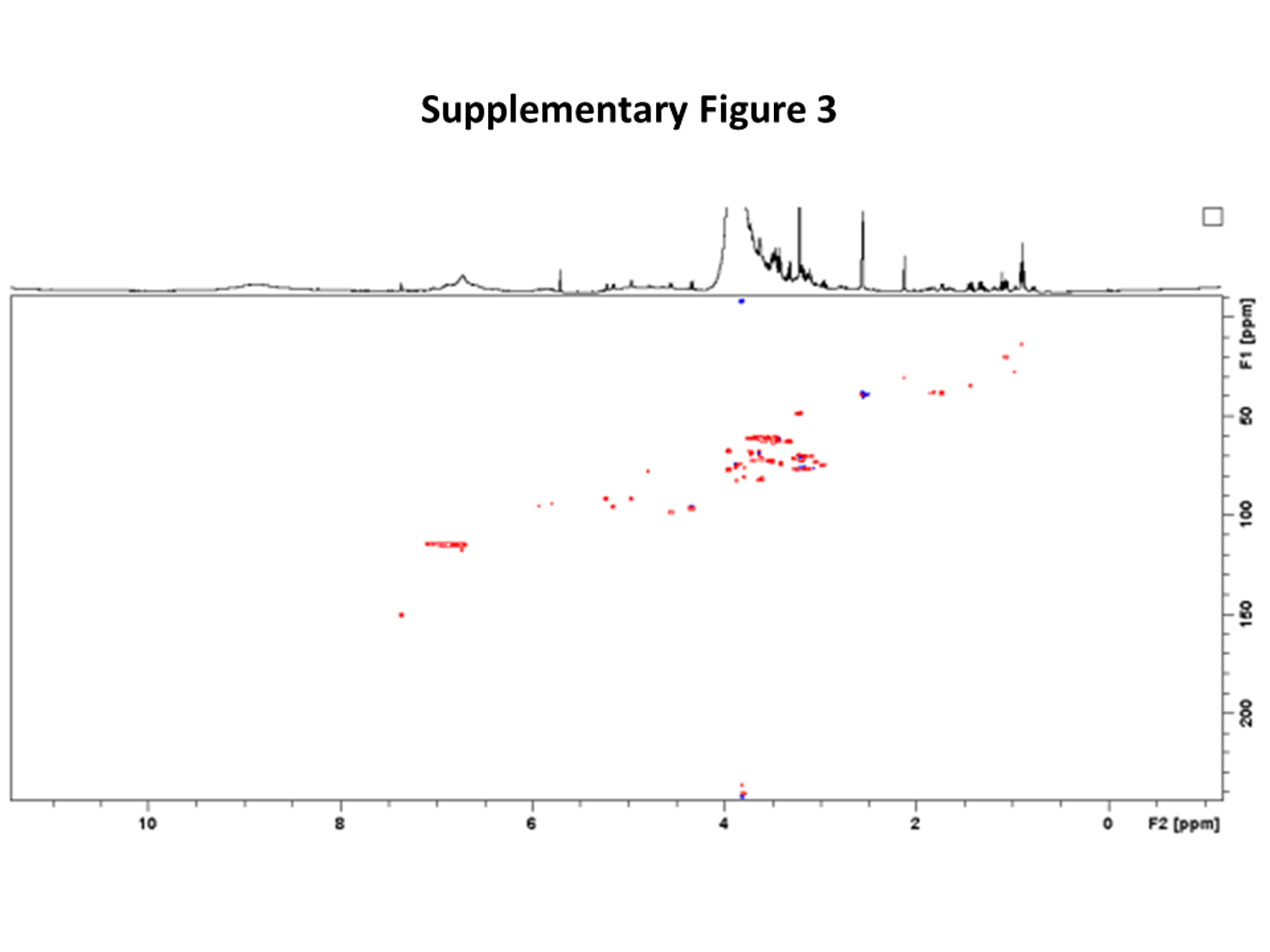

Supplement: Figure S3 — HSQC spectra of the BuOH fraction of U. tomentosa (400 MHz, DMSO-D6). (TIF) [file pone.0054618.s003.tif]

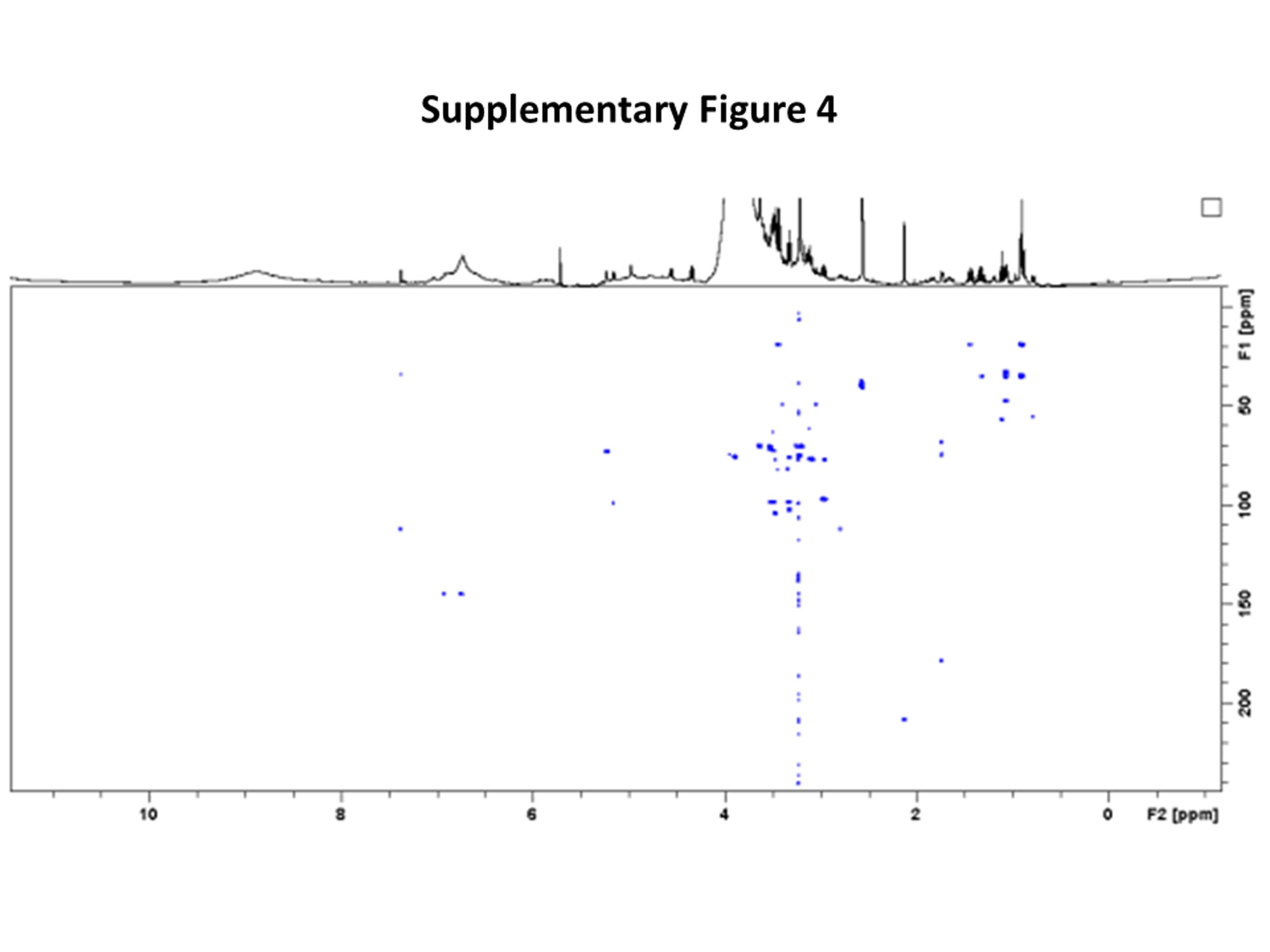

Supplement: Figure S4 — HMBC spectra of the BuOH fraction of U. tomentosa (400 MHz, DMSO-D6). (TIF) [file pone.0054618.s004.tif]

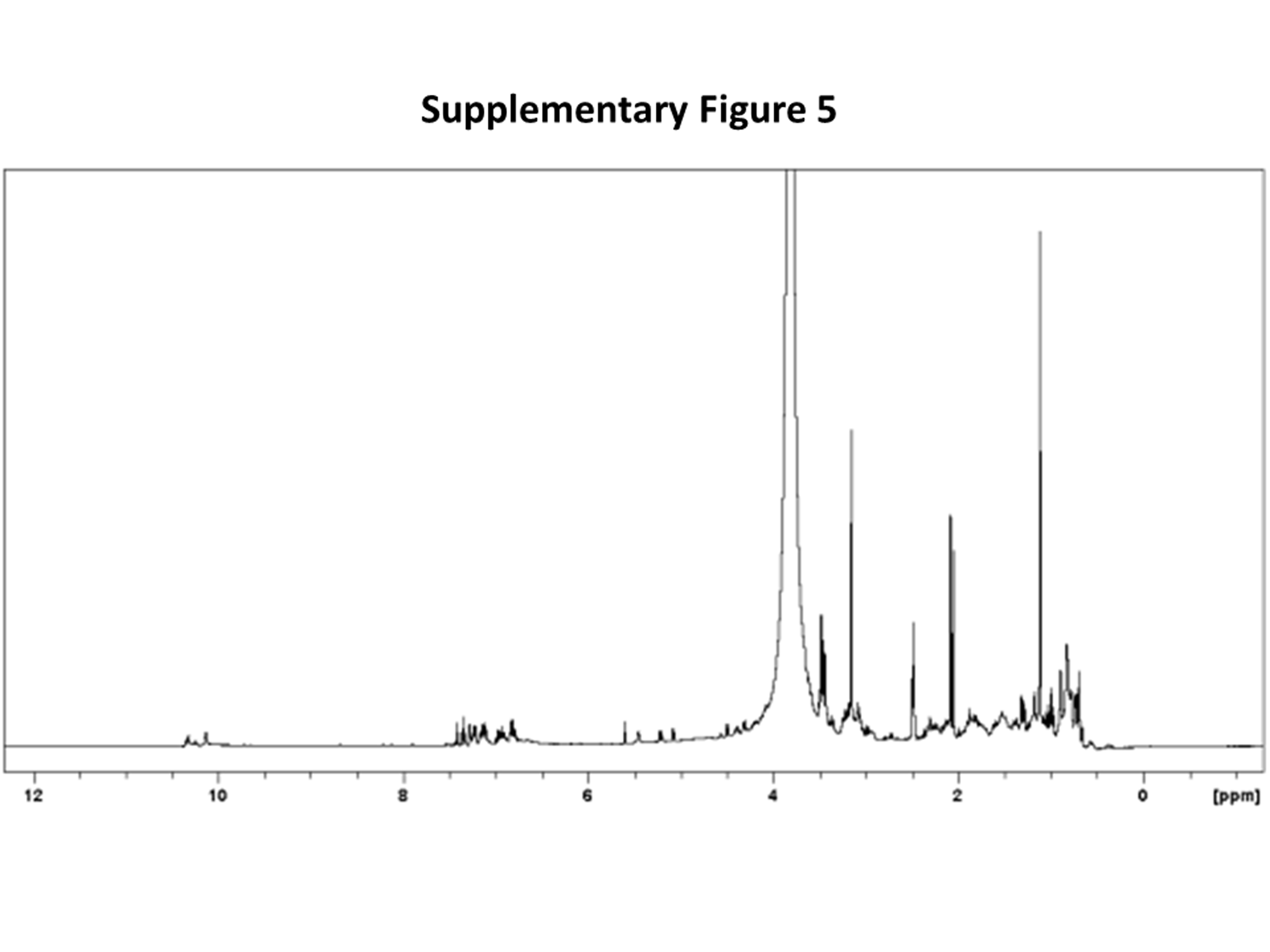

Supplement: Figure S5 — 1H NMR spectrum of the CHCl3 fraction of U. tomentosa (400 MHz, DMSO-D6). (TIF) [file pone.0054618.s005.tif]

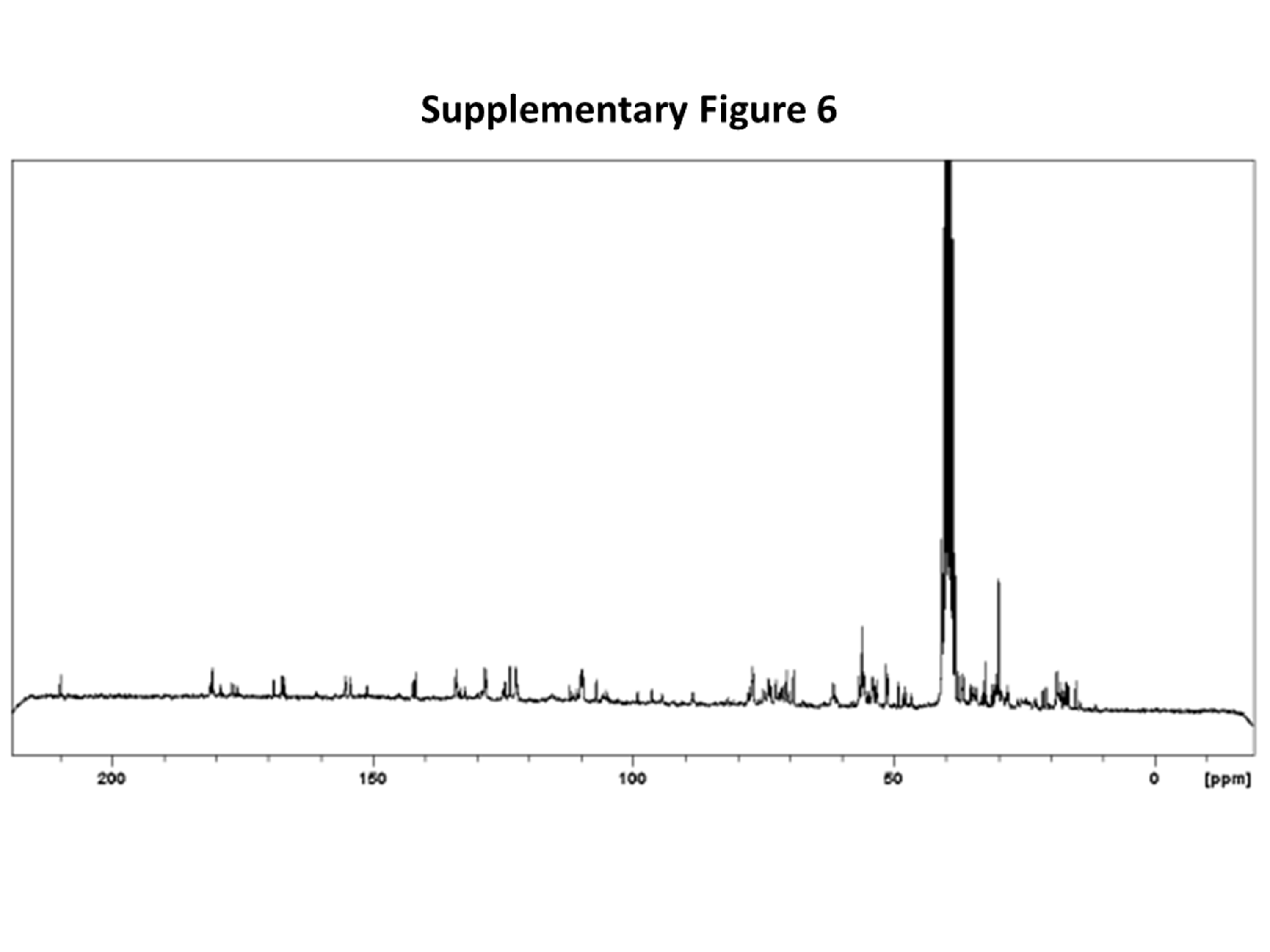

Supplement: Figure S6 — 13C NMR spectrum of the CHCl3 fraction of U. tomentosa (200 MHz, DMSO-D6). (TIF) [file pone.0054618.s006.tif]

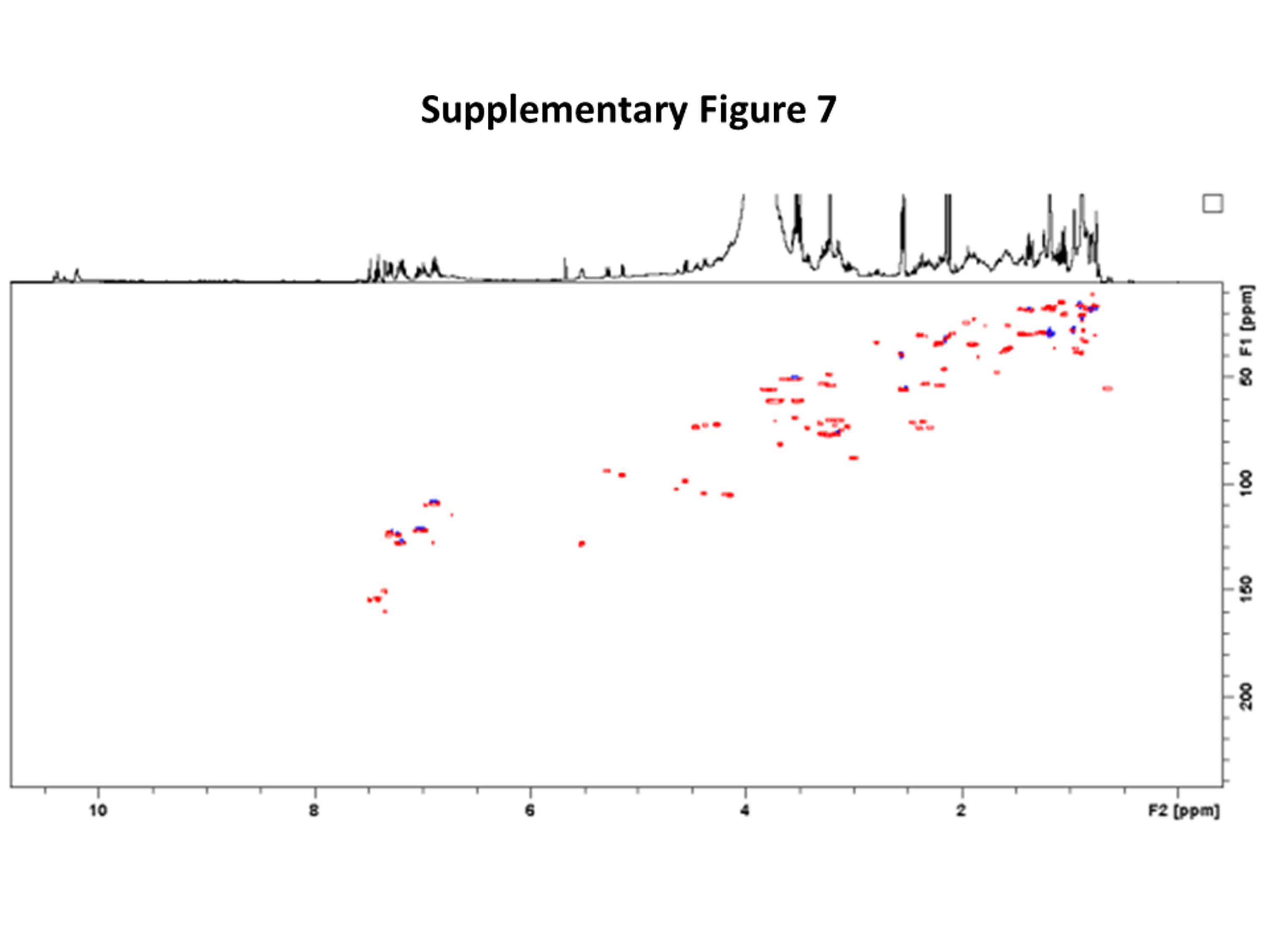

Supplement: Figure S7 — HSQC spectra of the CHCl3 fraction of U. tomentosa (400 MHz, DMSO-D6). (TIF) [file pone.0054618.s007.tif]

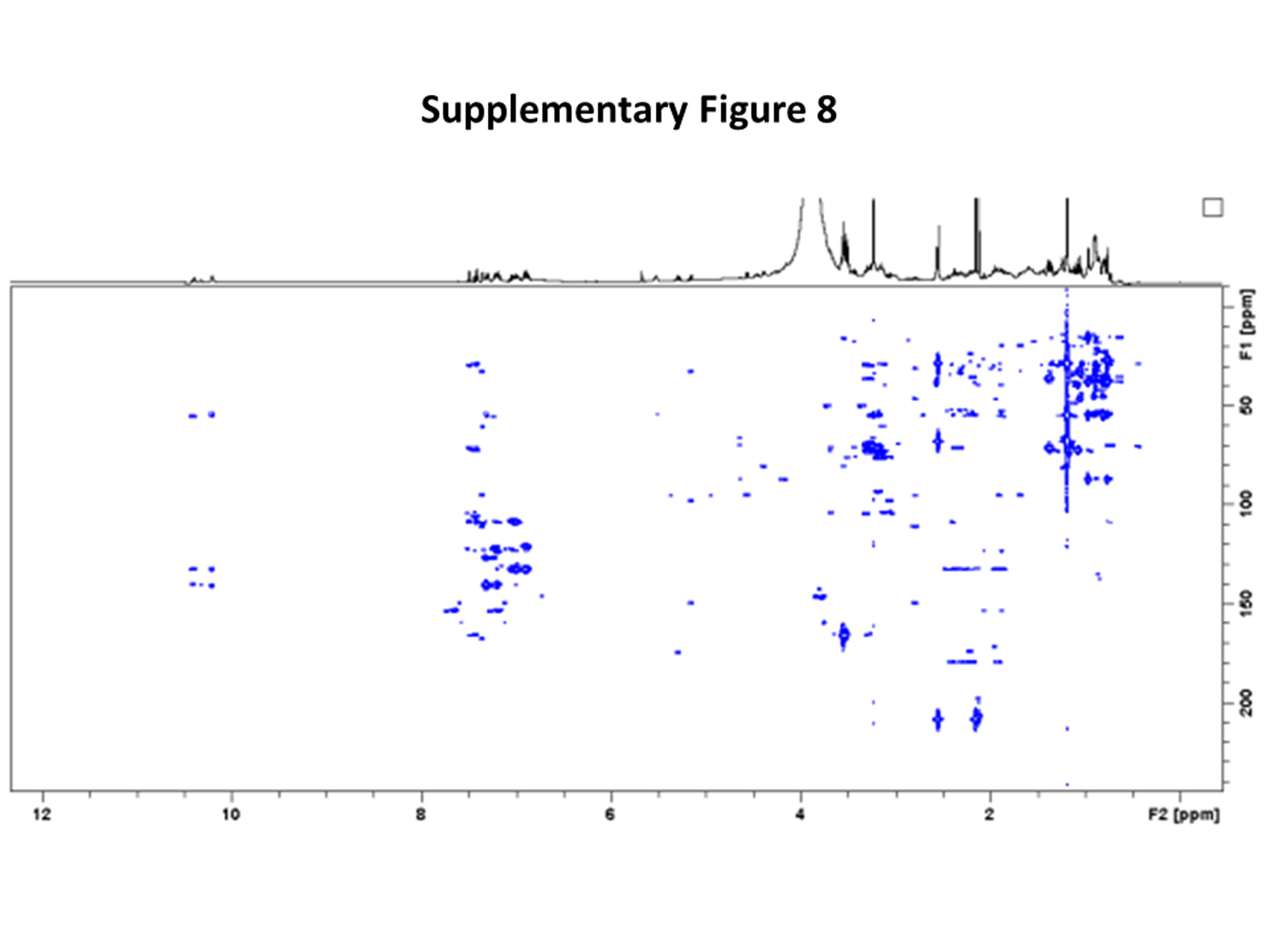

Supplement: Figure S8 — HMBC spectra of the CHCl3 fraction of U. tomentosa (400 MHz, DMSO-D6). (TIF) [file pone.0054618.s008.tif]
